# Advances in Endoscopic Management of Distal Biliary Stricture: Integrating Clinical Evidence into Patient-Specific Decision-Making

**DOI:** 10.3390/cancers17162644

**Published:** 2025-08-13

**Authors:** Reiko Yamada, Tetsuro Miwata, Yoshifumi Nakamura, Kenji Nose, Takamitsu Tanaka, Hirono Owa, Minako Urata, Yasuaki Shimada, Hayato Nakagawa

**Affiliations:** Department of Gastroenterology and Hepatology, Mie University Graduate School of Medicine, Tsu 514-8507, Japan; tetsurom56@med.mie-u.ac.jp (T.M.); yoshifumi-n@med.mie-u.ac.jp (Y.N.); kenji-nose@med.mie-u.ac.jp (K.N.); tanakatakamitsu@med.mie-u.ac.jp (T.T.); h-owa@med.mie-u.ac.jp (H.O.); m-urata@med.mie-u.ac.jp (M.U.); y-s-hayato@med.mie-u.ac.jp (Y.S.); nakagawah@med.mie-u.ac.jp (H.N.)

**Keywords:** distal biliary stricture, endoscopic ultrasonography-guided drainage, endoscopic retrograde cholangiopancreatography, percutaneous transhepatic biliary drainage, pancreatic adenocarcinoma, cholangiocarcinoma

## Abstract

The majority (70–85%) of biliary strictures—a narrowing of the bile ducts—are associated with malignancy, particularly pancreatic adenocarcinoma and cholangiocarcinoma, and are unresectable at presentation. Management options for distal biliary obstruction depend on several clinical factors, including the underlying cause and the location and complexity of the stricture. Endoscopic stent placement has lower morbidity and mortality rates compared with more invasive surgical options and is usually the first-line treatment to clear the blockage or allow the bile duct to drain internally. There are several stenting techniques to treat stenosis, but the current quality of evidence regarding the approach for different etiologies mostly ranges from moderate to low, and there is a lack of patient-specific guidelines regarding treatment decisions and for optimizing clinical outcomes. This review describes recent developments in stent technology and distal biliary stricture management, particularly endoscopic ultrasonography-guided drainage, and synthesizes findings from clinical trials and emerging research to highlight the role of patient-specific factors, such as anatomy and comorbidities, in tailoring treatment strategies. The integration of trial evidence into clinical practice paves the way for more effective and personalized care while addressing current knowledge gaps. Future research directions are identified to advance the development of innovative stent designs, enhance the quality of life, and improve long-term outcomes for patients with biliary strictures.

## Simple Summary

Biliary strictures, often caused by malignant conditions such as pancreatic and bile duct cancers, present a significant challenge in clinical care. These conditions frequently require minimally invasive treatments like endoscopic stent placement to restore bile flow and prevent complications. This review highlights recent advancements in endoscopic techniques and stent technologies for distal biliary strictures, such as endoscopic ultrasound-guided drainage and innovative stent designs, which improve outcomes for patients. While significant progress has been made, there is still a need for more personalized treatment approaches that consider individual patient factors, including anatomy, comorbidities, and disease progression. By integrating findings from recent clinical trials into practice, this review aims to guide clinicians in optimizing the management of biliary strictures, improving both patient quality of life and treatment success.

## 1. Introduction

Biliary strictures, defined as a narrowing of the bile ducts, are a significant clinical concern, particularly when associated with malignancies such as pancreatic adenocarcinoma and cholangiocarcinoma, which account for the majority (70–85%) of cases [1,2]. These conditions frequently present with unresectable disease, posing substantial challenges for effective management. Without intervention, chronic bile obstruction can lead to severe complications, including secondary biliary cirrhosis and liver failure [3]. Endoscopic stent placement has emerged as the preferred first-line treatment because of its superior safety profile and lower morbidity and mortality rates compared with more invasive surgical options [4,5].

Despite significant advancements in endoscopic techniques and stent technologies, the quality of evidence guiding clinical decisions is varied, often ranging from moderate to low [4,5]. Recent developments, such as endoscopic ultrasonography (EUS)-guided biliary drainage (EUS-BD), offer promising alternatives to traditional approaches like endoscopic retrograde cholangiopancreatography (ERCP) and percutaneous transhepatic biliary drainage (PTBD), particularly for patients with complex anatomy or challenging strictures [6,7]. However, integrating these advancements into clinical practice requires robust evidence from well-designed clinical trials, which are often lacking. Furthermore, current reviews fail to address how individualized patient factors, such as anatomy, disease progression, and comorbidities, can influence treatment outcomes [8,9].

This review presents an evaluation of recent and ongoing clinical trials that shape the understanding of biliary stricture management. By highlighting patient-specific considerations, such as the effect of comorbidities and anatomical variations on stent selection and procedural approaches, this review provides a comprehensive framework for optimizing the management of malignant distal biliary strictures.

## 2. Biliary Strictures and Current Management Options

### 2.1. Biliary Strictures

A biliary stricture is a narrowing of the bile ducts that obstructs the flow of bile from the liver to the intestines. Symptoms include jaundice, dark urine and pale stools, abdominal pain, pruritus, fatigue, weight loss, and cholangitis. Biliary strictures can result from benign and malignant causes [3]. The former include surgical injury (e.g., post-cholecystectomy), chronic pancreatitis, primary sclerosing cholangitis, gallstones, inflammation, and trauma to the bile ducts. However, the majority of biliary strictures are associated with malignancy [1]. The common causes of malignant distal biliary stricture are pancreatic adenocarcinoma, cholangiocarcinoma, ampullary carcinoma, and metastatic disease [2]. Before drainage, accurate differentiation between pancreatic adenocarcinoma, cholangiocarcinoma, ampullary carcinoma, metastatic disease, and benign biliary strictures is essential, as it directly influences both the treatment strategy and prognosis. Histological confirmation using EUS-guided tissue acquisition (EUS-TA) or transpapillary forceps biopsy should be pursued whenever feasible to establish a definitive diagnosis and avoid inappropriate management [10,11,12,13].

### 2.2. Current Management Options

There are a few management options for distal biliary obstruction, which depend on several clinical factors, namely the underlying cause and the location and complexity of the stricture. The first-line treatment is usually stent placement (ERCP or PTBD) to clear the blockage or to allow the bile duct to drain internally or externally. ERCP is commonly used to treat obstructions to relieve the blockage, and it is the primary palliative tool for biliary drainage of malignant biliary strictures. PTBD, in which a catheter is introduced through the skin and the liver parenchyma, is more invasive than ERCP and is used when ERCP is not possible, for example, when anatomical variations or tight strictures prevent access of the endoscope or successful cannulation [14].

EUS-BD, in which ultrasound is used to visualize the bile duct and guide a needle for drainage directly through the gastrointestinal wall into the bile duct, has emerged as an effective option compared with ERCP or PTBD, offering equivalent or improved clinical outcomes and reduced complications in patients with altered anatomy or malignant biliary obstruction [6,7].

Other management modalities include medical management, surgical intervention, and oncologic therapies. Medical management with antibiotics alone is effective only in cases of mild biliary infection where there is no significant obstruction, or when the obstruction is minor and likely to resolve on its own. Antibiotics can also serve as a bridge to more definitive drainage if the patient is too unstable for immediate procedures. Surgery (e.g., biliary bypass surgery or resection) is typically considered for biliary drainage only when endoscopic or percutaneous approaches are unsuitable or unsuccessful. For example, surgery may be required when the patient has an altered anatomy, a large tumor, or when the biliary obstruction is refractory to stent-based treatments. However, surgery is associated with a higher risk of complications, longer recovery time, and higher mortality rates compared with endoscopic procedures [15]. As most biliary strictures are associated with unresectable malignancy, biliary strictures are often managed in conjunction with biliary drainage and oncologic therapies to shrink the tumor and relieve biliary obstruction.

### 2.3. Clinical Decision-Making for Distal Biliary Stricture Treatment

Clinicians use a number of key guidelines to determine the appropriate treatment for distal biliary stricture, including (1) cause (benign or malignant) of the stricture; (2) patient symptom severity; (3) obstruction location (distal vs. proximal); (4) patient condition and comorbidities; and (5) response to previous treatments [4,8,9].

As well as the type of intervention, some clinical options may vary in the treatments, including the timing of intervention, the type of stent used, the placement of stents, and the material and size of stents [16], depending on factors such as the obstruction characteristics and the age, comorbidities, and life expectancy of the patient. Although there are reviews describing the management of biliary strictures [5,8,17,18,19,20,21,22,23,24,25,26], none of them provide comprehensive, patient-specific guidelines that update the existing management algorithms with the most recent advancements in endoscopic techniques and management strategies. In the following sections, we describe the recent advances in endoscopic techniques and stents and provide patient-specific recommendations to guide clinical decision-making for the treatment of biliary drainage.

## 3. Treatment of Biliary Strictures

Biliary stents relieve obstruction in the biliary tree or are used to treat biliary leaks. ERCP is considered the standard first-line treatment for biliary strictures [8]. Advantages of ERCP include its minimally invasive nature, high success rate, and ability to provide both diagnostic and therapeutic interventions, while limitations involve risks, such as pancreatitis, infection, bleeding, and perforation, and challenges in patients with altered anatomy or difficult-to-access strictures [20,27]. PTBD was often used as a salvage method when ERCP fails or in patients with altered anatomy or comorbidities who cannot tolerate surgical intervention [28]. Although biliary stenting is clinically effective in relieving both malignant and non-malignant obstructions, reintervention is often required owing to complications, such as stent migration, tumor ingrowth, and epithelial overgrowth, as well as internally from biofilm development and subsequent clogging [29].

### 3.1. Recent Advances in Stent Technology

There have been several recent developments in endoscopic biliary drainage management. In the early days of biliary drainage management with stents, plastic stents (PSs) were the standard option. PSs were cost-effective and relatively easy to place. However, their major limitation was the high rate of occlusion due to biofilm formation and biliary sludge, typically requiring replacement every 3–4 months.

Some of the most exciting advances in endoscopic biliary drainage management involve enhanced stent technology. The type, placement, construction material, shape, length, and diameter of the stent may all affect stent longevity and the treatment outcome [30,31]. Self-expandable metal stents (SEMSs) that adapt to the shape and diameter of the bile duct were introduced in the 1990s, offering a larger lumen and longer patency compared with PSs. This marked a significant advancement for patients, as it reduced the need for frequent stent replacements. Some older SEMSs contained ferromagnetic materials that could interfere with magnetic resonance imaging (MRI). However, most modern SEMSs are now manufactured using MRI-compatible materials, minimizing this concern in current clinical practice. SEMSs are associated with longer stent patency, lower reintervention rates, and longer patient survival [15]. While the unit cost of SEMSs is higher than that of PSs, the superior patency of SEMSs reduces the frequency of reinterventions, ultimately leading to lower overall treatment costs. Furthermore, biliary stents may have different shapes (e.g., straight or curved), lengths, and diameters. The size and shape of the stent will depend on the clinical scenario, as well as the opinion and skill of the operating endoscopist, but there are currently no comprehensive guidelines that address the choice of stent characteristics for the treatment of biliary strictures.

#### 3.1.1. Preoperative Drainage in Operable Cases

The selection of stents for preoperative biliary drainage in pancreatic cancer is based on current guidelines and recent research (Table 1). The Japanese, ESGE (European Society of Gastrointestinal Endoscopy) [32], and ASGE (American Society for Gastrointestinal Endoscopy) [33] guidelines all recommend the use of SEMSs for preoperative biliary drainage. The ESGE 2018 guidelines advise against routine preoperative drainage except in cases of cholangitis, severe symptomatic jaundice, delayed surgery, or neoadjuvant chemotherapy. The ASGE 2024 guidelines suggest SEMSs over PSs for distal malignant biliary obstruction, based on 15 randomized controlled trials.

In patients undergoing surgery without neoadjuvant chemotherapy, SEMSs are associated with lower rates of recurrent biliary obstruction (RBO) but higher complication rates, such as pancreatitis and cholecystitis [34,35]. In patients receiving neoadjuvant chemotherapy, SEMSs maintain longer patency but also pose concerns regarding adverse events [36,37,38]. However, the impact of SEMSs on the safety and efficacy of subsequent radiation therapy remains unclear due to a lack of sufficient evidence.

Alternative approaches are being investigated, for example, the potential advantages of thinner SEMSs (e.g., 6 mm) over the widely used 10 mm SEMSs, given their lower complication rates. The “PURPLE SIX STUDY” evaluated the safety and efficacy of 6 mm SEMSs for preoperative biliary drainage in patients with obstructive jaundice due to distal malignant biliary obstruction [39]. The results highlight that 6 mm SEMSs have comparable outcomes to previously reported PSs regarding technical success and adverse event rates, but show a significant advantage in maintaining patency and reducing the need for additional interventions before surgery. The authors suggest that 6 mm SEMSs may be preferable for preoperative biliary drainage.

**Table 1:** Key prospective trials on preoperative biliary drainage in pancreatic cancer.

Study	Patient Group	Primary Outcome	Stent Type	Sample Size	AE Rate	RBO Rate
**Without Neoadjuvant Chemotherapy**						
Tol et al., 2016 [34]	Resectable pancreatic cancer	PBD-related complications	10 Fr PSs	102	20%	6%
			10 mm FCSEMSs	53	11%	30%
Song et al., 2016 [35]	Resectable pancreatic cancer	Rate of PBD procedure-related AEs prompting additional intervention	10 Fr PSs	43	5%	16%
			10 mm CSEMSs	43	12%	5%
Mandai et al., 2022 [40]	Resectable pancreatic cancer	Endoscopic reintervention rate during the waiting period for surgery	10 Fr PSs	35	9%	29%
			10 mm FCSEMSs	2	25%	0%
**With Neoadjuvant Chemotherapy**						
Gardner et al., 2016 [36]	Resectable and borderline resectable pancreatic cancer	Time to stent occlusion, attempted surgical resection, or death after the initiation of neoadjuvant therapy	10 Fr PSs	21	0%	52%
			10 mm UCSEMSs	17	18%	35%
			10 mm FCSEMSs	16	25%	25%
Seo et al., 2019 [37]	Resectable and borderline resectable pancreatic cancer	Sustained biliary drainage	8–10 mm UCSEMSs	60	24%	27%
			8–10 mm FCSEMSs	59	20%	28%
Tamura et al., 2021 [38]	Borderline resectable pancreatic cancer	Rate of stent dysfunction until surgery or tumor progression	10 Fr PSs	11	63.6%	72.8%
			10 mm FCSEMSs	11	18.2%	18.2%

AE: adverse event; FCSEMSs: fully covered self-expandable stents; PBD: preoperative biliary drainage; PSs: plastic stents; UCSEMSs: uncovered self-expandable metal stents.

#### 3.1.2. PSs vs. SEMS in Surgically Unresectable Cases

The ESGE (2018) guidelines strongly recommend SEMSs for palliative drainage of malignant extrahepatic biliary obstruction and recommend against uncovered SEMSs (UCSEMSs) for drainage when malignancy is unconfirmed [32]. The ASGE (2024) guidelines suggest covered SEMSs for distal malignant biliary obstruction and recommend against UCSEMSs in patients with distal biliary obstruction from a pancreatic mass when malignancy is unconfirmed [33].

Findings from clinical trials comparing PSs and SEMSs indicate the advantages of SEMSs (Table 2), including longer patency and reduced need for reintervention [41,42]. However, a large multicenter study noted adverse events associated with SEMS, indicating the need for a careful risk-benefit assessment in clinical practice [43].

#### 3.1.3. Covered and Uncovered Stents for Surgically Unresectable Cases

An important advance is the development of fully covered self-expandable metal stents (FCSEMSs), which are covered with a synthetic covering to minimize tumor ingrowth [50,51,52]. FCSEMSs last longer and have lower occlusion rates than plastic or uncovered stents [53,54]. The incidence of stent migration is lower when using FCSEMSs compared with PSs, and anchoring fins may further reduce stent migration [55].

Although the advent of FCSEMSs has led to improved outcomes in some clinical scenarios, there is still controversy about the optimal stent cover. One systematic review and meta-analysis of covered vs. uncovered SEMSs for malignant distal biliary strictures concluded that although there was a risk reduction of ~32% for both stent failure and patient mortality with FCSEMSs, this difference was not significant [56]. Migration and sludge rates were higher with covered SEMS, whereas tumor ingrowth was more likely with UCSEMSs. Overall, the authors concluded that there was no added benefit of covered SEMSs compared with UCSEMSs. However, a more recent randomized controlled trial (RCT) meta-analysis of covered versus uncovered metal stents concluded that covered SEMSs are superior to UCSEMSs in the prevention of recurrent biliary obstruction (RBO) in patients with malignant distal biliary obstruction, particularly those caused by pancreatic cancer [44,45,57]. A recent systematic review and meta-analysis [54] comparing fully covered versus partially covered SEMSs for palliation of distal malignant biliary obstruction showed that partially covered SEMSs exhibited longer times to RBO than FCSEMSs with no difference in adverse events.

#### 3.1.4. Stent Diameter

Another important clinical decision involves the diameter of the stent. Smaller-diameter stents may be necessary for tight or complicated strictures, but they may affect sludge formation. When deciding between a smaller or larger-diameter stent, a number of factors must be considered:

- Risk of pancreatitis: Smaller-diameter stents, such as 6 Mm SEMS, are associated with a reduced risk of post-ERCP pancreatitis compared with larger-diameter stents, likely owing to less compression of the pancreatic duct.
- Risk of migration: Smaller-diameter stents may have a slightly higher risk of migration; however, proper positioning and stent selection can mitigate this risk.

The choice of stent diameter should balance these factors. For preoperative cases, 6 Mm SEMSs may be optimal, while for palliative or long-term cases, larger stents may be more suitable. Tailoring the choice to the patient’s specific clinical scenario is essential. Clinical trials into stent materials and diameters are shown in Table 1 and Table 2.

**Figure 1:**
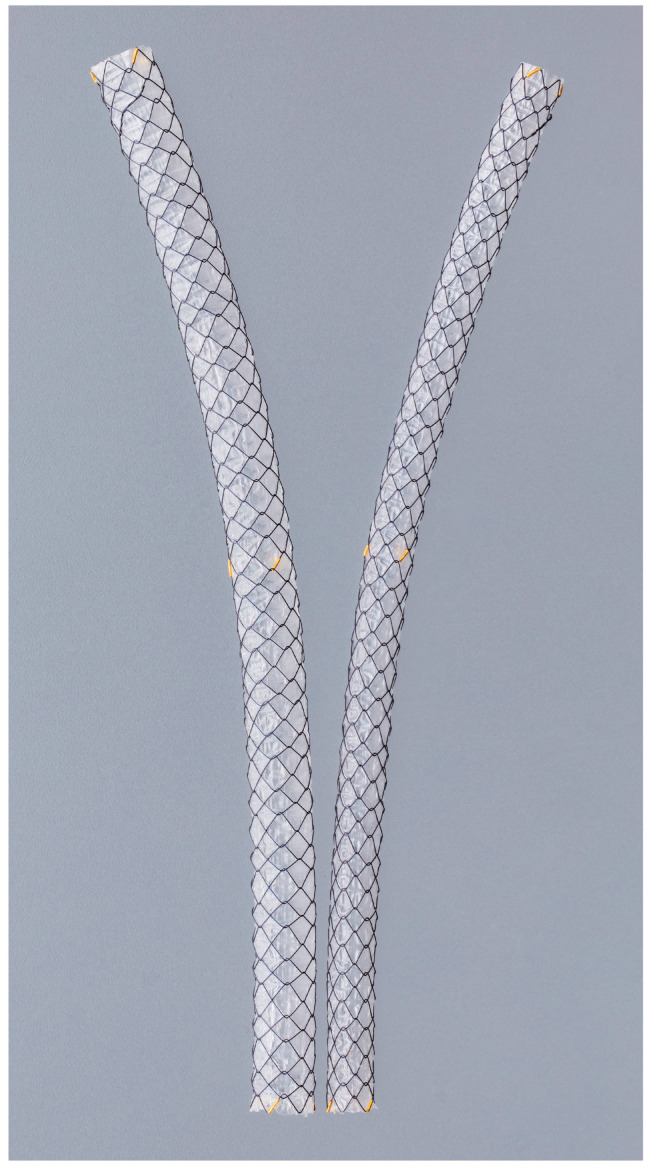
Representative image of fully covered self-expandable metal stents (FCSEMSs) with 6 mm and 8 mm diameters. These thinner stents are increasingly used in clinical trials and practice due to their potential advantages in reducing procedure-related adverse events while maintaining adequate biliary drainage. © 2025 Boston Scientific Corporation. All rights reserved.

Mukai et al. [48] compared FCSEMSs with 10 and 12 mm diameters for unresectable malignant distal biliary obstructions and showed that 12 mm FCSEMSs provided a longer time to RBO (TRBO) compared with 10 mm FCSEMSs. However, a multicenter prospective study of patients with distal malignant biliary obstruction comparing 8 and 10 mm diameter FCSEMSs showed that there was no difference in TRBO, survival, and adverse events using 8 mm diameter stents compared with 10 mm stents [47]. In a retrospective study of patients with 6 or 10 mm diameter FCSEMSs to treat distal malignant biliary obstruction, the 6 mm FCSEMSs had a cumulative incidence of RBO comparable to that of the 10 mm FCSEMSs and fewer stent-related adverse events [58]. Representative images of 6 Mm and 8 mm fully covered self-expandable metal stents (FCSEMSs) are shown in Figure 1.

An ongoing trial—the COSMIC UNISON trial—is an RCT investigating the effect of FCSEMSs diameter (6 mm vs. 10 mm) on the TRBO following stent placement by ERCP for unresectable malignant distal bile duct stricture patients [59].

#### 3.1.5. Emerging Stent Technologies

Further advancements in stent technology include drug-eluting stents, such as those used in the MIRA III trial (NCT02460432). This trial compared drug-eluting SEMSs with traditional covered SEMSs, highlighting their potential to significantly enhance patency and reduce occlusion rates while addressing some limitations of FCSEMSs [60]. Other studies included a trial evaluating plastic stent anchoring to reduce migration (NCT03439020). By using a plastic stent to anchor FCSEMSs, the migration rates were effectively reduced without compromising the stent’s patency or efficacy [61].

Another recent advance is the multi-hole SEMS, which was investigated in a recent trial (NCT05786326). This stent design includes multiple small side holes in the stent membrane, with the aim of preventing obstruction of bile duct branches and reducing migration risks through ingrowth stabilization [62]. In patients with unresectable malignant distal biliary obstruction, multi-hole SEMSs provided the longest stent patency time with a lower RBO rate compared with conventional SEMS, a lower stent migration rate than FCSEMSs, and a lower tumor ingrowth rate than UCSEMSs [63].

Ascending cholangitis is a common complication of biliary stents. Anti-reflux stents have a valve that prevents duodenal content reflux into the bile ducts, which reduces the risk of ascending cholangitis. They are particularly useful in patients with long-term stents or those at high risk of infection [17]. Biliary stenting of distal malignant biliary obstruction using duckbill-shaped anti-reflux metal stents has been shown to be feasible and safe for biliary drainage and to achieve an acceptable TRBO [64]. However, the benefits and efficacy of anti-reflux stents are not entirely clear yet, and further research regarding the construction, shape, and placement is required [17,65].

### 3.2. EUS-BD

EUS-BD is an emerging technique that involves accessing the biliary tree with a fine-needle aspiration needle and guidewire to create an anastomosis tract with cautery and/or dilation and deploying a stent under endosonographic and fluoroscopic visualization [66]. EUS-guided techniques include antegrade and transmural stenting. In EUS-guided antegrade stenting, a stent is placed from the bile duct to the intestine via a guidewire passed through the stricture in an antegrade direction. This technique is used when access to the stricture via ERCP is difficult, especially in cases of altered anatomy. In EUS-guided transmural stenting, a stent is placed directly through the stomach or duodenum into the bile duct and is useful for biliary drainage in cases of distal obstruction where ERCP is not feasible or fails [67,68,69].

Although EUS-BD and ERCP have a similar safety and efficacy profile in the treatment of malignant biliary obstruction, EUS-BD is associated with a lower risk of reintervention and adverse events than ERCP [70,71]. Thus, recent evidence suggests that EUS-BD is not only an effective salvage option following failed ERCP, but also a feasible and safe primary drainage modality in selected patients with malignant distal biliary obstruction [72,73,74,75,76,77]. Table 3. shows key RCTs of EUS-BD vs. ERCP-BD or PTBD for malignant biliary obstruction.

While EUS-BD is generally safe, it is technically demanding and associated with a high risk of adverse events (10–30%). Thus, it should be performed with caution. Common complications include bile leakage, bleeding, pneumoperitoneum, peritonitis, and stent migration. However, recent advances have significantly enhanced the safety and efficacy of EUS-BD, and it may be beneficial in patients with altered anatomy or an inaccessible papilla. A systematic review of six studies concluded that EUS-BD is associated with fewer reinterventions and subsequent adverse events than PTBD [27]. In addition, RCTs comparing PTBD and EUS-BD have also demonstrated the efficacy of EUS-BD [78,79]. EUS-BD is increasingly recommended in certain patient groups, such as those with surgically altered anatomy or gastrointestinal obstruction that makes it difficult to access the papilla [4,80,81].

#### 3.2.1. EUS-CDS

**Figure 2:**
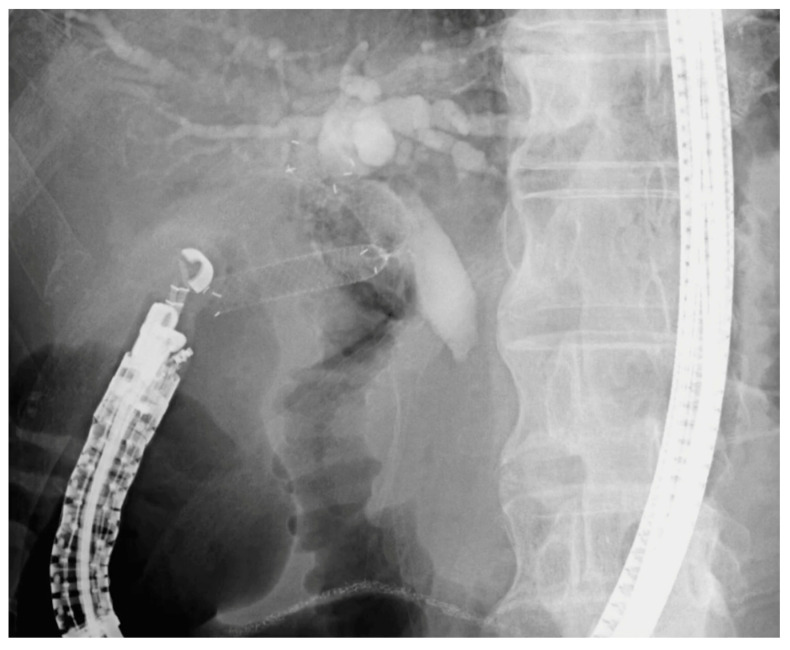
Fluoroscopic image of EUS-guided choledochoduodenostomy (EUS-CDS). A fully covered self-expandable metal stent is deployed from the duodenal bulb into the common bile duct under endosonographic and fluoroscopic guidance for biliary drainage in a patient with malignant distal biliary obstruction.

EUS-guided choledochoduodenostomy (EUS-CDS) is an endoscopic technique for internal biliary drainage in patients with distal malignant biliary obstruction when ERCP fails or is not feasible. Under EUS guidance, the dilated extrahepatic bile duct (usually the common bile duct) is punctured from the duodenal bulb, followed by tract dilation and deployment of SEMSs to establish a choledochoduodenal anastomosis (Figure 2). Several clinical studies and meta-analyses have evaluated the efficacy and safety of EUS-CDS, reporting technical success rates of 90–100% and clinical success rates of 85–95% [81,82].

**Figure 3:**
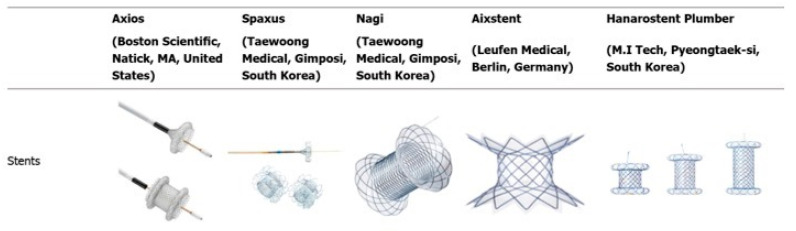
Types of commercially available lumen-apposing metal stents. This figure illustrates the structural differences among various LAMS devices, including the AXIOS (Boston Scientific), SPAXUS (Taewoong Medical), NAGI (Taewoong Medical), HOT AXIOS (Boston Scientific), and HANAROSTENT (M.I. Tech). Modified from Sharma P et al. Alternative uses of lumen apposing metal stents. World J Gastroenterol. 2020; 26 (21):2715–2732 [83]. Distributed under the Creative Commons Attribution-NonCommercial 4.0 International (CC BY-NC 4.0) license.

Although lumen-apposing metal stents (LAMSs), characterized by a barbell shape with bilateral flanged ends, are not yet approved for use in Japan, several reports have suggested their utility in EUS-CDS (Figure 3). This design is believed to contribute to a reduced risk of stent migration. The DRA-MBO Trial (NCT03000855) compared EUS-CDS using LAMSs with ERCP and covered metal stents in patients with malignant distal biliary obstruction. EUS-CDS demonstrated shorter procedural times, higher technical success rates, and comparable 1-year stent patency rates to conventional stents [76].

The ELEMENT trial (NCT03870386) directly compared EUS-CDS using LAMSs and ERCP in patients with malignant biliary obstruction. Conducted across multiple centers in Canada, the study demonstrated that EUS-CDS was not inferior to ERCP in terms of technical and clinical success rates, while also showing a lower incidence of complications, such as cholangitis and post-procedural pancreatitis [77]. Furthermore, EUS-CDS has been associated with significantly shorter hospital stays and lower overall treatment costs, highlighting its potential as a preferred option for biliary drainage in cases where ERCP has failed, provided appropriate expertise is available [82,84]. The BAMPI trial (NCT04595058) assesses the clinical benefits of combining coaxial double-pigtail PSs with LAMSs in EUS-CDS. Early findings from this trial suggest that this approach reduces recurrent biliary obstruction rates, enhances stent patency, and minimizes the need for reinterventions, addressing limitations of single-stenting techniques and offering more durable therapeutic solutions [85].

#### 3.2.2. EUS-HGS

EUS-guided hepaticogastrostomy (EUS-HGS) involves the creation of an anastomosis tract between the left intrahepatic bile ducts and the stomach under EUS guidance, enabling the placement of a stent to achieve internal biliary drainage. EUS-HGS is a pivotal technique for biliary drainage in patients with malignant biliary obstruction, particularly when conventional ERCP fails. Clinical studies report technical success rates of EUS-HGS ranging from 90% to 100%, with clinical relief of biliary obstruction achieved in 70–90% of cases [86,87,88,89].

**Figure 4:**
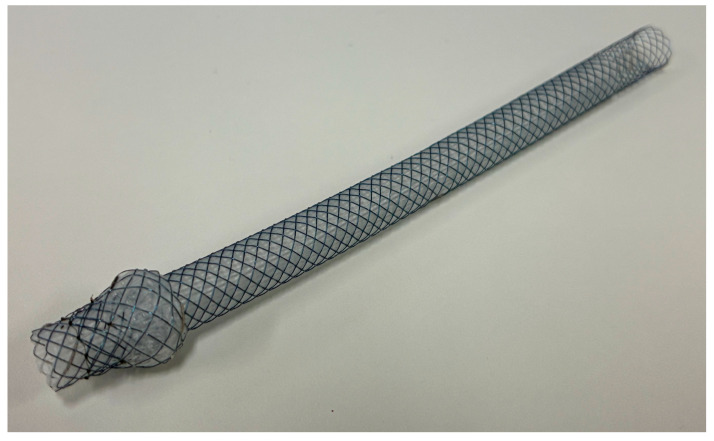
Representative image of “Spring Stopper Stents”. © 2025 Century Medical Corporation. All rights reserved.

**Figure 5:**
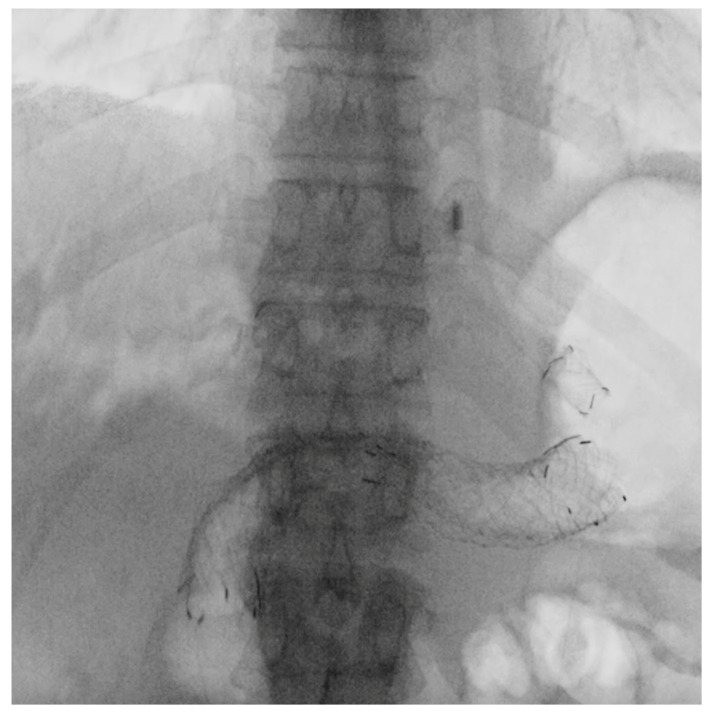
Fluoroscopic image of EUS-guided hepaticogastrostomy (EUS-HGS). A partially covered self-expandable metal stent, “Spring Stopper Stents”, is placed between the left intrahepatic bile duct and the stomach in a patient with malignant biliary obstruction and an inaccessible papilla.

Although the optimal stent type for EUS-HGS has not been established, several reports are available. One such report suggests that the use of PSs in EUS-HGS has a better safety profile and comparable patency to metal stents [90]. To decrease the risk of stent migration into the abdominal cavity, partially covered SEMSs with a spring-like anchor on the gastric side, known as Spring Stopper Stents, have been developed [91]. Currently, Spring Stopper Stents are the only reimbursable option in Japan (Figure 4 and Figure 5). A newly designed, partially covered laser-cut stent with anti-migration anchoring hooks and a thin tapered tip (7.2F), called a Hook stent (Zeon Medical), has been developed to prevent serious adverse events associated with EUS-HGS [92].

Another recently described innovation is antegrade stent placement across malignant distal biliary obstructions followed by EUS-HGS (EUS-HGAS), which creates two biliary drainage routes [93]. Although there was no difference in survival between groups treated with EUS-HGS and EUS-HGAS, the TRBO was significantly longer in the HGAS group (716 days) than in the HGS group (194 days).

The development of access tools and delivery systems has streamlined the procedure and minimized risks associated with EUS-HGS. Furthermore, EUS-HGS has demonstrated longer stent patency and reduced need for reintervention compared with PTBD, making it an increasingly preferred option when ERCP fails.

## 4. A Patient-Specific and Evidence-Based Approach to Biliary Stricture Management

### 4.1. The Need for Personalized Treatment Strategies

Clinical decision-making should consider the presence, absence, and size of masses in or near the bile duct, as well as the resectability of the lesion [4]. Although ERCP remains the standard first-line therapy for malignant biliary obstruction, EUS-BD is gaining recognition for providing superior outcomes in specific patient populations, particularly in cases involving altered anatomy or failed ERCP [4,8,94].

Recent trials, such as CARPEGIEM (NCT06375967) and NCT04898777, are actively evaluating how anatomical considerations and tumor location inform the selection between EUS-GBD, EUS-CDS, and ERCP [95,96]. However, the overall quality of evidence remains moderate to low in many clinical scenarios [4,5], highlighting the need for updated, individualized guidelines that incorporate patient-specific data and recent technological innovations.

### 4.2. Integration of Evidence into Clinical Practice

The translation of clinical trial findings into real-world practice is essential for improving outcomes in biliary stricture management. Recent trials have demonstrated incremental advancements that improve specific aspects of patient management and open new avenues for managing malignant biliary obstructions. Table 4 summarizes the relevant trials, offering an overview of their contributions and insights into their application in practice. Innovations in stent design have further enhanced the efficacy of biliary drainage. Another significant advancement is the growing use of EUS-BD as an alternative to ERCP in cases of malignant biliary obstruction.

The integration of trial findings into clinical workflows ensures that patients benefit from innovations while addressing practical challenges, such as resource limitations, procedural complexities, and patient-specific factors. Robust long-term studies are needed to evaluate outcomes, such as cost-effectiveness, reintervention rates, and survival across diverse patient groups.

## 5. Conclusions

Recent and ongoing clinical trials continue to redefine the management of distal biliary strictures, emphasizing the importance of patient-specific strategies. Advances, particularly in EUS-BD techniques and innovative stent designs, have demonstrated significant potential to optimize clinical outcomes, reduce complications, and enhance the quality of life for affected patients. Despite these advancements, a critical need remains for comprehensive, patient-specific guidelines to integrate trial findings into routine clinical decision-making. Future research should focus on bridging the gap between emerging evidence and real-world practice by incorporating individual patient factors into treatment strategies.

## Figures and Tables

**Table 2:** Key randomized controlled trials on stent selection for unresectable distal malignant biliary obstruction.

Study	Journal	Study Design	Stent Type	Median TRBO (days)	Median Stent Patency
Davids et al., 1992 [41]	Lancet	Prospective RCT	9F PSs vs. 8–10 mm SEMSs	NR	126 vs. 273 days
Soderlund & Linder, 2006 [42]	Gastrointest Endosc	Prospective RCT	10F PSs vs. SEMSs	NR	1.8 vs. 3.6 months
Isayama et al., 2004 [44]	Gut	Prospective randomized study	10 mm FCSEMSs vs. 10 mm UCSEMSs	304 vs. 166	225 vs. 193 days
Kitano et al., 2013 [45]	Am J Gastroenterology	Prospective randomized study	10 mm FCSEMSs vs. 10 mm UCSEMSs	187 vs. 132	219 vs. 167 days
Loew et al., 2009 [46]	Gastrointest Endosc	Prospective RCT	Nitinol 6- and 10 mm Zilver stents, and 10 mm stainless steel Wallstent	115, 111, and 103 days	143, 186, and 187 days
Kawashima et al., 2019 [47]	Dig Endosc	Prospective study	8 mm vs. 10 mm FCSEMS	275 vs. 293	NR
Mukai et al., 2024 [48]	Gastrointest Endosc	Prospective randomized trial	12 mm vs. 10 mm covered SEMSs	172 vs. 120	NR
Hasegawa et al., 2024 [49]	Gastrointest Endosc	RCT	Laser-cut vs. braided SEMSs	220 vs. 418	NR

FCSEMSs: fully covered self-expandable metal stents; UCSEMSs: uncovered self-expandable metal stents; NR: not reported; SEMSs: self-expandable metal stents; RCT: randomized controlled trial; TRBO: time to recurrent biliary obstruction.

**Table 3:** Key randomized controlled trials of EUS-BD vs. ERCP-BD/PTBD/for malignant biliary obstruction.

Study	Year	Country	Situation of the Study	Stent Used	Group	Patient (*n*)	Technical Success Rate (%)	Clinical Success Rate (%)	Median Follow-Up (days)	Reintervention Rate (%)	Stent Patency Rate (%)	Stent Patency Time (Days)	Adverse Events (%)
Artifon et al. [78]	2012	Brazil	After a failed ERCP	Self-expanding metal stents	EUS-BD/PTBD	13/12	100/100	100/100	80/75	NR/NR	NR/NR	NR/NR	15.3/25.0
Lee et al. [79]	2016	Korea	After a failed ERCP	Self-expandable metal stent	EUS-BD/PTBD	34/32	94.1/96.9	87.5/87.1	≥90/≥90	32.4/87.5	NR/NR	NR/NR	8.8/31.2
Bang et al. [72]	2018	USA	Primary treatment	Self-expandable metal stent	EUS-BD/ERCP	33/34	90.9/94.1	97.0/91.2	190/174	3.0/2.9	NR/NR	188/197 (median)	21.2/14.7
Paik et al. [74]	2018	Korea	Primary treatment	Self-expandable metallic stent	EUS-BD/ERCP	64/61	93.8/90.2	90.0/94.5	144/165	15.6/42.6	85.1/48.9 (at 6 months)	NR/NR	6.3/19.7
Park et al. [75]	2018	Korea	Primary treatment	Self-expandable metal stent	EUS-BD/ERCP	14/14	92.8/100	100/92.8	95/147	15.4/30.8	NR/NR	379/403 (median)	0/0
Zhao et al. [73]	2022	China	Primary treatment	Metal biliary stent	EUS-AG/ERCP	28/30	100/96.67	NR/NR	NR/NR	0/3.3	NR/NR	252/241 (median)	3.57/26.67
Teoh et al. [76]	2023	International	Primary treatment	Lumen-apposing metal stent	EUS-CDS/ERCP	79/76	96.2/76.3	93.7/90.8	365/365	11.3/12.7	91.1/88.1 (at 1 year)	183.2/161.3 (mean)	16.5/17.1
Chen et al. [77]	2023	Canada and France	Primary treatment	Lumen-apposing metal stent	EUS-CDS/ERCP	73/71	90.4/83.1	84.9/85.9	NR/NR	9.6/9.9	NR/NR	163.9/200.1 (mean)	12.3/12.7

**Table 4:** Summary of cited clinical trials and their contributions to practice.

Registration Number and Status (as of January 2025)	Focus Area	Aims and/or Findings	Insights into Clinical Practice
NCT03439020 [61] Completed Results Published	Anchoring techniques to reduce stent migration	Plastic stent anchoring significantly reduced migration rates in FCSEMSs without compromising patency	Highlights the importance of anchoring mechanisms to improve stent stability in high-risk scenarios
NCT05786326 [62] Completed	Multi-hole SEMSs	Evaluated the performance of multi-hole SEMSs in preventing bile duct branch obstruction and reducing migration risks	Highlights advanced stent designs for long-term management
NCT05595122 (SCORPION-II-p) [97] Results In-Press	Advanced FCSEMS designs	Enhanced patency and reduced tumor ingrowth	Improves outcomes in distal malignant biliary obstruction
NCT02460432 (MIRA III) [60] Completed Results Published	Drug-eluting vs. covered SEMSs	Demonstrated prolonged patency and reduced tumor ingrowth with drug-eluting SEMSs	Supports stent selection tailored to obstruction type and long-term therapeutic goals
NCT03000855 [76] Completed Results Published	DRA-MBO Trial: EUS-BD vs. ERCP	Demonstrated shorter procedural times and higher technical success with EUS compared with ERCP, with comparable 1-year stent patency	Supports EUS as an effective alternative to ERCP in advanced malignancy cases
NCT03870386 (ELEMENT) [77] Completed Results Published	EUS-BD vs. ERCP	Demonstrated non-inferiority of EUS-BD to ERCP with reduced complications and shorter hospital stays	Reinforces EUS as a viable alternative to ERCP, particularly in challenging biliary obstructions
NCT04595058 (BAMPI) [85] Completed Results Published	LAMSs with coaxial PSs	Enhanced stent patency, reduced recurrent obstruction, and fewer reinterventions	Refines strategies for durable biliary drainage in malignant obstructions
NCT06375967 (CARPEGIEM) [95] Recruiting	First-line palliative EUS-GBD vs. EUS-CDS	Evaluates biliary drainage strategies for first-line palliative care	Highlights the role of EUS in palliative management
NCT04898777 [96] Completed	EUS-CDS vs. ERCP	Compared EUS-CDS with ERCP for drainage outcomes	Reinforces the role of EUS in improving drainage outcomes
NCT03812250 [98] Completed	EUS-BD vs. ERCP for malignant obstruction	Evaluated drainage outcomes and reintervention rates with EUS-BD compared with ERCP	Highlights EUS-BD’s potential as a preferred approach in anatomically challenging cases.
NCT06196164 [99] Recruiting	EUS-BD vs. ERCP for low malignancy obstructions	Compares technical success and quality-of-life outcomes	Provides insights into optimizing palliative drainage
NCT02103413 [100] Completed	EUS-BD vs. PTBD for failed ERCP	Investigated the technical success and complication rates of EUS-BD compared with PTBD	Potential of EUS-BD as a minimally invasive alternative in cases with anatomical challenges

ERCP: endoscopic retrograde cholangiopancreatography; EUS-BD: EUS-guided biliary drainage; EUS-CDS: EUS-guided choledochoduodenostomy; EUS-GBD: EUS-guided gallbladder drainage; FCSEMSs: fully covered self-expandable metal stents; LAMSs; lumen-apposing metal stents; SEMSs: self-expandable metal stents.

## Abbreviations

The following abbreviations are used in this manuscript:

EUS	Endoscopic ultrasonography
ERCP	Endoscopic retrograde cholangiopancreatography
PTBD	Percutaneous transhepatic biliary drainage
EUS-BD	EUS-guided biliary drainage
FCSEMSs	Fully covered self-expandable metal stents
SEMSs	Self-expandable metal stents
PSs	Plastic stents
ESGE	European Society of Gastrointestinal Endoscopy
ASGE	American Society for Gastrointestinal Endoscopy
RBO	Recurrent biliary obstruction
TRBO	Time to RBO
RCT	Randomized controlled trial
EUS-CDS	EUS-guided choledochoduodenostomy
LAMSs	Lumen-apposing metal stents
EUS-HGS	EUS-guided hepaticogastrostomy
EUS-GBD	EUS-guided gallbladder drainage
HCC	Hepatocellular carcinoma
HIFU	High-intensity focused ultrasound
RFA	Radiofrequency ablation
